# A novel constructed *SPT15* mutagenesis library of *Saccharomyces cerevisiae* by using gTME technique for enhanced ethanol production

**DOI:** 10.1186/s13568-017-0400-7

**Published:** 2017-06-02

**Authors:** Ashraf A. M. M. El-Rotail, Liang Zhang, Youran Li, Shuang Ping Liu, Gui Yang Shi

**Affiliations:** 1 The Key Laboratory of Industrial Biotechnology, Ministry of Education, National Engineering Laboratory for Cereal Fermentation Technology, School of Biotechnology, Jiangnan University, 1800 Lihu Road, Wuxi, 214122 Jiangsu China; 2 Faculty of Environmental Agricultural Science, El Arish University, El Arish, North Sinai 45526 Egypt

**Keywords:** Bioethanol, Error-prone PCR, Ethanol production, Ethanol tolerance, Global transcription machinery engineering, *SPT15*

## Abstract

During the last few years, the global transcription machinery engineering (gTME) technique has gained more attention as an effective approach for the construction of novel mutants. Genetic strategies (molecular biology methods) were utilized to get mutational for both genes (*SPT15* and *TAF23*) basically existed in the *Saccharomyces cerevisiae* genome via screening the gTME approach in order to obtain a new mutant *S. cerevisiae* diploid strain. The vector pYX212 was utilized to transform these genes into the control diploid strain *S. cerevisiae* through the process of mating between haploids control strains *S. cerevisiae* (MAT-a [CICC 1374]) and (MAT-α [CICC 31144]), by using the oligonucleotide primers SPT15-*Eco*RI-FW/SPT15-*Sal*I-RV and TAF23-*Sal*I-FW/TAF23-*Nhe*I-RV, respectively. The resultant mutants were examined for a series of stability tests. This study showed how strong the effect of using strategic gTME with the importance of the modification we conducted in Error Prone PCR protocol by increasing MnCl_2_ concentration instead of MgCl_2_. More than ninety mutants we obtained in this study were distinguished by a high level production of bio-ethanol as compared to the diploid control strain.

## Introduction

Ethanol (ETOH) is a colorless liquid produced by the fermentation of sugars by a variety of microbes such as *Saccharomyces cerevisiae* (*S. cerevisiae*), *Escherichia coli* (*E. coli*), *Klebsiella oxytoca* and *Zymomonas mobilis* (Tan et al. 2016). It is used as fuel, solvent, sterilizer, antiseptic for wounds, cosmetics and pharmaceutical industry. *S. cerevisiae* has been studied extensively as a model organism among eukaryotes (Botstein and Fink 1988). The requirements of *S. cerevisiae* to produce a high level of ethanol are its ability to withstand an elevated concentration of ethanol and sugar existed in the fermentation medium (Hou 2009).

Recently, the global transcription machinery engineering (gTME) has been applied as an effective technique to enhance the target specific phenotype of microbes (Alper et al. 2006). The gTME uses molecular biology methods such as Error-prone polymerase chain reaction (Ep-PCR), DNA shuffling, etc. to construct an initial transcription factor and screen the target phenotype to obtain the enhanced metabolic flux or bacteria with a specific phenotype. Ep-PCR is a fast and cheap molecular biology method for random mutation in a particular piece of DNA. This technique is a based on PCR and its reaction mixture composition. Upon replication in EP-PCR, Taq polymerase is modulated by alteration of the composition of the reaction buffer usually by adjusting Mg and Mn ions as well as dNTPs in the PCR reaction. In this condition, the Taq polymerase, which lack DNA proof-reading property, introduces errors in a newly synthesized DNA during base pairing. The control of PCR reaction composition regulates the frequency of mis-incorporation of nucleotide bases into the sequence. In some directed evolution experiments, a high number of mutations around 9–12 bp/kb may be obtained (Cadwell and Joyce 1992). Therefore, cloning of the resulted PCR fragment or screened libraries of mutants provide proteins with desired activity (McCullum et al. 2010; Drummond et al. 2005). It produces most of the functional genes; however RNA polymerase II transcription efficiency is determined by initiation of transcription factors and promoter binding protein, and one of the first transcription factors in *S. cerevisiae* is TATA-binding protein (TBP). The *SPT15* regulates the expression of most of all those genes, and essentially its variants modulate many genes those are necessary for ethanol tolerance. Previous research showed that variants of the *S. cerevisiae* TBP *SPT15* could improve the yield of ethanol (Tan et al. 2016) by using the gTME (Alper et al. 2006).

The protocols established by (Cadwell and Joyce 1992; Alper et al. 2006) were adopted on our study with some modification in the process of polymerase chain reaction (PCR) protocol. Different concentrations of MnCl_2_ were used instead of MgCl_2_ to produce mutations in the sequence of *SPT15* and *TAF23* genes by using the Ep-PCR amplification process. This approach was satisfactorily applied to get mutants by which it would be able to improve ethanol production and ethanol tolerance.

## Materials and methods

### Microbial strains and culture media

*Escherichia coli* JM109 was used as a host for plasmid construction. It was grown in Luria–Bertani (LB) medium containing 5 g/L yeast extract, 10 g/L Bacto-peptone and 10 g/L (NaCl) and cultured at 37 °C. Super optimal broth (SOB) containing 5 g/L yeast extract, 20 g/L Bacto-peptone, 0.95 g/L (MgCl_2_), 0.186 g/L (KCl) and 0.5 g/L (NaCl) was used before spreading on solid media after transformation. *S. cerevisiae* strain was used for genetic manipulation and cultured in Basic medium-Bacto-peptone glucose broth (YPD) medium; this medium was used for routine growth of yeast strains at 30 °C and contained 10 g/L yeast extract, 20 g/L Bacto-peptone and 20 g/L glucose. Solid media contained 2% agar for both types of mediums. Respective antibiotics [ampicillin (Amp) 100 mg/L; kanamycin (Kan) 35 mg/L] were added to maintain the plasmids (Liu et al. 2014). For screening, the medium (YPDG) containing 10 g/L yeast extract, 20 g/L Bacto-peptone, 20 g/L glucose and resistance gene Glycosid-418 (G418) that used with the final concentrations (250 and 350 µg/mL), respectively (Wang et al. 2004). The Fermentation medium (YPDT) was contained 10 g/L yeast extract, 20 g/L Bacto-peptone, 20 g/L glucose and 0.2 g/L Thiamine.

#### Genetic manipulation strain

*Saccharomyces cerevisiae* eukaryotic system containing the non-mutant genes chromosomal copy of *SPT15* and *TAF23* genes was selected. Table 1 shows that two haploids types of industrial ethanol-producing yeast (*MAT*-a/*MAT*-*α*) were mated and genetically manipulated in this study, as well Table 2 shows that oligonucleotide primers (*MAT*-F), (*MAT*-a) and (*MAT*-alpha) were used in order to obtain a strain possessing diploid genetic traits, named as *S. cerevisiae* R-control. The protocol of Hoffman and Winston (1987) was used to extract genomic DNA (gDNA) with slight modifications by using Mini-DNA fragment Rapid Kit (BioSCi Biotech Co., LTD, Hangzhou, China).

**Table 1:** Microbial strains and plasmids used

Strains/plasmids	Relevant characteristics	Source of reference
Strains		
*S. cerevisiae* haploid strain	Wild-type (*MAT*- *α*) CICC 1374	China center of industrial culture collection (CICC)
*S. cerevisiae* haploid strain	Wild-type (*MAT*- a) CICC 31144	
*S. cerevisiae* R-control	Diploid strain	This study
*E. coli* JM109	*recA1 supE44 endA1 hsdR17 gyrA96 relA1 thi (Lac*-*proAB) F’[traD36 proAB* ^+^ *lacI* ^*q*^ *lacZM15]*	Stratagene
Plasmids		
pMD19-T vector	*Amp* ^*r*^ clone vector	TaKaRa, Japan
pYX212	*Amp* ^*r*^ TPI promoter	This study
pYX212-kan-*SPT15*-*Mu*	pYX212 with *SPT15* mutant gene	This study
pYX212-kan-*TAF23*-*Mu*	pYX212 with *TAF23* mutant gene	This study

*Amp* ^r^: ampicillin resistant, Kan: kanamycin

**Table 2:** The oligonucleotide primers used

Primer names	Sequence (5′–3′)^a^
SPT15-*Eco*RI-FW	CCG*GAATTC*ATGGCCGATGAGGAACGTTTAAAGG
SPT15-*Sal*I-RV	CGCTAG*GTCGAC*TCACATTTTTCTAAATTCACTTAGCACA
TAF23-*Nhe*I-RV	ACTCGA*GCTAGCC*TAACGATAAAAGTCTGGGCGACCT
TAF23-*Sal*I-FW	CGCTAG*GTCGAC*ATGGATTTTGAGGAAGATTACGAT
pYX212-FW	GGGCAGCATAATTTAGGAG
pYX212-RV	AGGGATGTAT CGGTCAGTCA
425-TT-*Bam*HI-FW	CGC*GGATCC*ATCCGTATGATGTGCCTGACTA
426-TT-*Eco*RV-RV	AATAAGA*TATCAG*GGAAGAAAGCGAAAGGAG
(*MAT*-*F*)	AGTCACATCAAGATCGTTTATGG
(*MAT*-a)	ACTCCACTTCAAGTAAGAGTTTG
(*MAT*-*alpha*)	GCACGGAATATGGGACTACTTCG

^a^Restriction sites are in italic and underlined

### Target genes

TATA-binding protein subunit of transcription factor complexes Transcription factor II D (TFIID) and Transcription factor II B (TFIIB) were used (Cormack and Struhl 1992). It plays an enormous role during gene expression in yeast cells (Hampsey 1998). Additionally, much gene regulation takes place by targeting TBP with coactivators and corepressors, which interact with TBP for gene transcription (Lewis and Reinberg 2003). Mutations in the TBP confer enhanced tolerance to stress in *S. cerevisiae* of ethanol and glucose (Lee and Young 1998). The *SPT15* and *TAF23* were used in this study to improve cellular phenotype for yeast strain with ethanol tolerance. Both genes express via transcription during RNA polymerase II coupled with the association of TBP with general transcription factors and regulators (Alper and Stephanopoulos 2007; Gietz and Woods 2006; Jungwoo et al. 2011).

#### Extraction, cloning and overexpression of the target genes

The extracted amount of gDNA was used as a template with sequence-specific primers mentioned below in Table 2 to begin the process of cloning of *SPT15* and *TAF23* genes in this study. Standard methods were used for PCR amplification, analysis of DNA fragments and electroporation as directed by Green and Sambrook (2012). The extension reaction was adjusted on 72 °C for 10 min. In addition to, the annealing temperature was changed it to 58, 55 °C for *SPT15* and *TAF23* genes, respectively. The PCR and Ep-PCR experiments were performed by using the Real-Time PCR Detection System with software CFX Manager 3.0 (Bio-Rad, USA), using rTaq DNA polymerase (TaKaRa, Japan). All PCR products were sequenced to verify the mutation rate for all the new mutations and to get the mutant genes sequence for *SPT15*-*Mu* and *TAF23*-*Mu*.

This was followed by cloning and transformation and then plated the ligation product on specific antibiotic-containing medium. The plasmid(s) and gene(s) verification was done by Sangon Biotech CO., LTD, Shanghai, China.

### Design of primers and vectors

#### Plasmid design with target genes

The oligonucleotide primers SPT15-*EcoR*I-FW/SPT15-*Sal*I-RV and TAF23-*Sal*I-FW/TAF23-*Nhe*I-RV were designed as primers for *SPT15* and *TAF23* genes, respectively. The genome sequence of *S. cerevisiae* and both genes sequences were searched using the *S. cerevisiae* Genome Database and the National Center for Biotechnology Information. All the target genes were PCR amplified from the gDNA of *S. cerevisiae* R-control as a template using oligonucleotide primers SPT15-*EcoR*I-FW/SPT15-*Sal*I-RV and TAF23-*Sal*I-FW/TAF23-*Nhe*I-RV, respectively as referred in Table 2. The resulting PCR products were purified by Mini-DNA fragment Rapid Kit and the target genes were ligated with vector (pYX212) which formed two plasmids pYX212-Amp-SPT15 and pYX212-Amp-TAF23. Both plasmids were individually transformed into *E. coli* JM109 competent cells for multiplication on SOB medium with Amp (100 μg/mL) at 37 °C for 24 h. Then, these plasmids (pYX212-Amp-SPT15 and pYX212-Amp-TAF23) were extracted and purified by using a Plasmid Mini-Preps Kit (BioSCi Biotech Co., LTD, Hangzhou, China). These genes obtained from the previous step were used as a template to initiate DNA amplification using Ep-PCR technique for the next steps and preserved in pMD19-T vector.

#### Plasmid design with mutant genes

**Fig. 1:**
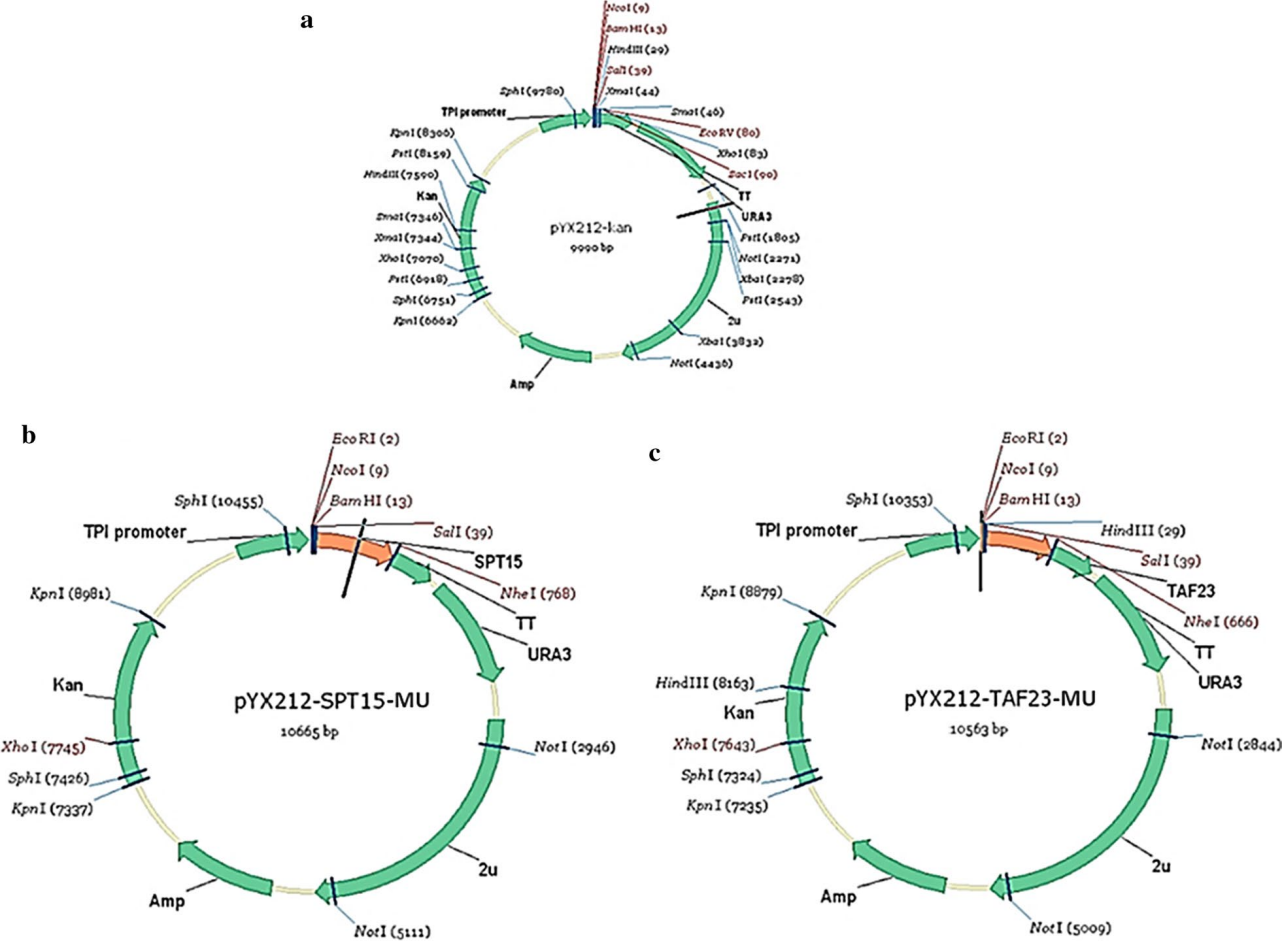
The physical maps of recombinant plasmids. **a** The pYX212-kan (empty vector). **b**, **c** The pYX212 with *SPT15*-*Mu* and *TAF23*-*Mu*, respectively. The genes are under the R-control of TPI promoter and terminator with *Eco*RI and *Sal*I restriction sites for *SPT15* and *Sal*I and *Nhe*I restriction sites for *TAF23*; (Amp^r^) ampicillin resistance gene; (Kan^r^) Kanamycin resistance gene; (G418) resistance gene

The pYX212-TPI plasmid was designed as an expression vector; designed as bacterial and yeast expressing system. For this plasmid, the prokaryotic and eukaryotic selection markers are Amp, uracil and Kan as shown in Fig. 1. Oligonucleotide primers were designed based on the pYX212 vector, with restriction sites *EcoR*I and *Sal*I for *SPT15* gene mutagenesis; moreover *Sal*I and *Nhe*I were used for *TAF23*. For plasmid construction, overexpression of genes and electroporation, standard methods were applied as directed (Green and Sambrook 2012; Lee et al. 2007) (Table 2).

Both *SPT15* and *TAF23* genes were randomly mutated, amplified by standard PCR and ligated individually with pYX212 plasmid. The fragment of *SPT15* mutant gene and pYX212 plasmid were digested with *EcoR*I and *Sal*I, however the pYX212 plasmid and *TAF23* gene were digested by using restriction enzymes *Sal*I/*Nhe*I and ligated by using T4 DNA Ligase (Thermo Scientific CO., LTD, Meridian, USA) to get pYX212-kan-*SPT15*-*Mutant* (pYX212-kan-*SPT15*-*Mu*) and pYX212-kan-*TAF23*-*Mutant* (pYX212-kan-*TAF23*-*Mu*) plasmids, respectively.

### Construction of mutant genes libraries

The gTME strategy and Ep-PCR were used to express *SPT15* and *TAF23* genes encoding TBP. They are commonly used method to give the irregular variations of mutations into a defined segment and specific of the DNA (Hou et al. 2015). Those approaches are based on the amplification of the entire gene(s) using primers that possess the region of interest (Hemsley et al. 1989). Those techniques were used to create randomized genomic libraries by working on the principle r-Taq polymerase that can anneal incompatible base pairs (bp) during PCR (Drummond et al. 2005; McCullum et al. 2010). The latter produced the largest possible number of changes in the gene sequence (low, medium and high) with strong and stable rate of mutation(s). The procedure was adjusted by adding 1–30 μL of MnCl_2_ (5 mM), and mixed with dNTPs one by one and retain 22 μL of MgCl_2_ (25 mM) within 100 μL Ep-PCR reaction mixture.

Accordingly, this modification allowed initiating DNA amplification beginning with small amounts of original molecule to bring to considerable amounts of a high random mutagenesis. Then, the library of mutations was constructed with that combination of genes *SPT15*-*Mu* and *TAF23*-*Mu*. The aforesaid mutations were inserted into the strain by using pYX212 vector. The new mutations could induce the production of ethanol at high rate which is expected to be higher than the R-control strain.

The mutagenic reaction mixture contained different amount of MnCl_2_ (1, 3, 5, 10, 20 and 30 μL) (5 mM), 22 μL of MgCl_2_ (25 mM), 10 µL of (10× Taq Buffer), 1 µL (each) PCR primers (mentioned above in Table 2), 1 µL of Template gDNA, 1 µL rTaq DNA polymerase, 10 µL of (10× dNTP Mix), then fulfill the volume mixture to 100 µL of distilled water. The (10× dNTP Mix) mixture was prepared by using an equal dNTP concentrations as follows: 2 µL of dATP (0.2 mM), 2 µL of dGTP (0.2 mM), 10 µL dCTP (1 mM), 10 µL dTTP (1 mM) and the distilled water was added until the volume of 100 µL. The dGTP, dNTP, dCTP, dTTP, and rTaq DNA polymerase enzymes were purchased from TaKaRa, Japan.

### Transformation for *SPT15*-*Mu* and *TAF23*-*Mu* genes into *S. cerevisiae*

High-efficiency yeast transformation procedure was determined according to the methods of Gietz (2014) and Gietz and Woods (2006), with some modifications. Briefly, each 50 μL plasmid harboring DNA mutant gene was added individually to a tube. Then the mixture was filled up to 380 μL alongside by using transformation mixture [240 µL PEG (50%), 36 µL LiAc (1 M), 50 µL ssDNA (2 mg/mL), 54 µL sterile distilled water]. total mixture was subsequently heated shockingly at 42 °C for 25 min. Cells were harvested by centrifugation at 2.4*g* for 2 min at room temperature, re-suspended in 1 mL sterile water, then mixed by inverting the tube up and down. 100 µL of the suspension was plated on agar medium containing G418 (100 µg/mL). The plates were incubated at 30 °C for 2 days for growth.

### Study of mutations characteristics

#### Mutations stability

Ten colonies were selected for the screening as a random mutation. In order to ensure the stability test of each colony, 100 µL was plated on YPD solid medium in triplicate with different concentration of Kan. The colony that has a good viability among the grown colonies was then transferred on YPD containing G418 (250 µg/mL) and incubated at 30 °C until single colonies appeared. By the unaided eye, the best five colonies were picked up after photographed the plates. Subsequently, re-grown on a fresh YPDG medium containing G418 (350 µg/mL); then incubated at 30 °C for 2 days. Afterward, the resistant colonies were streaked out (Guo et al. 2011; Zhang et al. 2011). Finally, colony PCR reaction was performed to verify the success of the mutations by using oligonucleotide primers 425-TT-*Bam*HI-FW/426-TT-*Eco*RV-RV as referred in Table 2. The PCR products were loaded on 1.5% Agarose and visualized to make sure the criteria of the stab le genes selection.

#### Ethanol tolerance of mutant strains

Ethanol tolerance test was used in order to assay the ability of the 102 mutations that harboring *SPT15* genes. It was carried out by spot assay in duplicate onto YPD plates containing Kan within various concentrations of ethanol 1, 3, 5, 7, 10 and 15% (v/v), respectively. The colonies forming ability and viability on a high level of ethanol were monitored after incubation at 30 °C for 3 days (Kasavi et al. 2014).

### Effect of ethanol concentration on the growth rate of mutant strains

The ethanol tolerance of the *SPT15* mutants and *S. cerevisiae* R-control strains were grown in 24 deep holes microplate containing 5 mL YPD medium in the absence of ethanol in each hole, as a primary pre-culture to grow the strains. The OD_595_ was reached 0.05, then 40 µL of the pre-cultures from *SPT15* mutants and R-control strain were inoculated into 24 deep holes microplate containing 3 mL liquid fresh medium present/absent of G418 (250 µg/mL). In this estimation, five various concentration of ethanol 0, 1, 3, 5 and 7% (v/v) were used for each treatment. Finally, mutants and R-control strain growth rate were monitored by measuring dry cell weight (DCW) and OD_595_ values. Microplate reader (EZ Read 800, Biochrom Ltd., Cambridge, UK), with Galapagos Exert Software (Version 1.1) were used to recording OD_595_ values.

### Measurement of the ethanol and glucose yield

#### Fermentation conditions

Fermentation was carried out in the cap-covered flasks (500 mL) containing 50 mL YPDT medium as followed: 20 g/L glucose, 10 g/L yeast extract, 20 g/L Bacto-peptone and 0.2 g/L thiamine (vitamin B1) (Kotarska et al. 2006; Xu et al. 2012), and 5 mL of inoculum were added. The mixture was cultured at 30 °C and 200 rpm till OD_595_ reached 1.

#### High performance liquid chromatography analysis

The broth samples were centrifuged at 1*g* for 20 min then filtered through a 0.22 μm filter (Whatman^®^ Spartan^®^ HPLC certified syringe filters, sterile, diam. 13 mm). The supernatant was appropriately diluted by 10% of Trichloro acetic acid (TDA) (1:1) for the product analysis. The concentrations of the samples were estimated by high performance liquid chromatography (HPLC) analysis, with a Shodex RI SUGER SH-1011 HPLC column (7 µm, 8 I.D. × 300 mm) (Showa Denko Co., Ltd., K.K., Japan). The column temperature was heated at 50 °C with 0.01 M H_2_SO_4_ as a mobile phase and flow rate was 0.8 mL/min. The concentrations were subsequently detected with a refractive index detector with 285 nm wave length (HITACHI High Technologies Co., Ltd., Tokyo, Japan) Model (CM 5110/5210/5310/5430/5450) to estimate the percentage of both glucose and ethanol in the samples. The HPLC analysis was performed by isocratic condition using 1% Sulfuric acid in water (v/v) as mobile phase. The product was eluted around 22 min for total running time per sample. All HPLC data were analyzed with EZChrom Elite (Version 3.3.2) SP2 Chromatography Data System, Agilent Tech software. The percentage of the ethanol yield production between the mutant strains and R-control strain was estimated by using the following equation:

$$ IR = [(EP_{ 2} /EP_{ 1} )*100] - 100 $$

IR increase of rate, EP_1_: ethanol production for mutant strains, EP_2_: ethanol production for R-control strain.

## Results

### Hybridization, amplification and construction of plasmid

The colonies were chosen for mating process between the haploid strains of *S. cerevisiae* (*MAT*-a and *MAT*-*α*) for the industrial ethanol-producing yeast. Conjunction process resulted a strain of *S. cerevisiae* possessing diploid genetic traits, and was named *S. cerevisiae* R-control. The gDNA of *S. cerevisiae* R-control was used as a template for PCR reaction. The clear bands of 723 and 621 bp for *SPT15* and *TAF23* genes, respectively were identified. Construction and ligation of plasmid with genes have resulted in clear bands at 3323 and 3221 bp for pMD19-T-Amp-SPT15 and pMD19-T-Amp-TAF23, respectively.

### Construction of mutant genes libraries

**Fig. 2:**
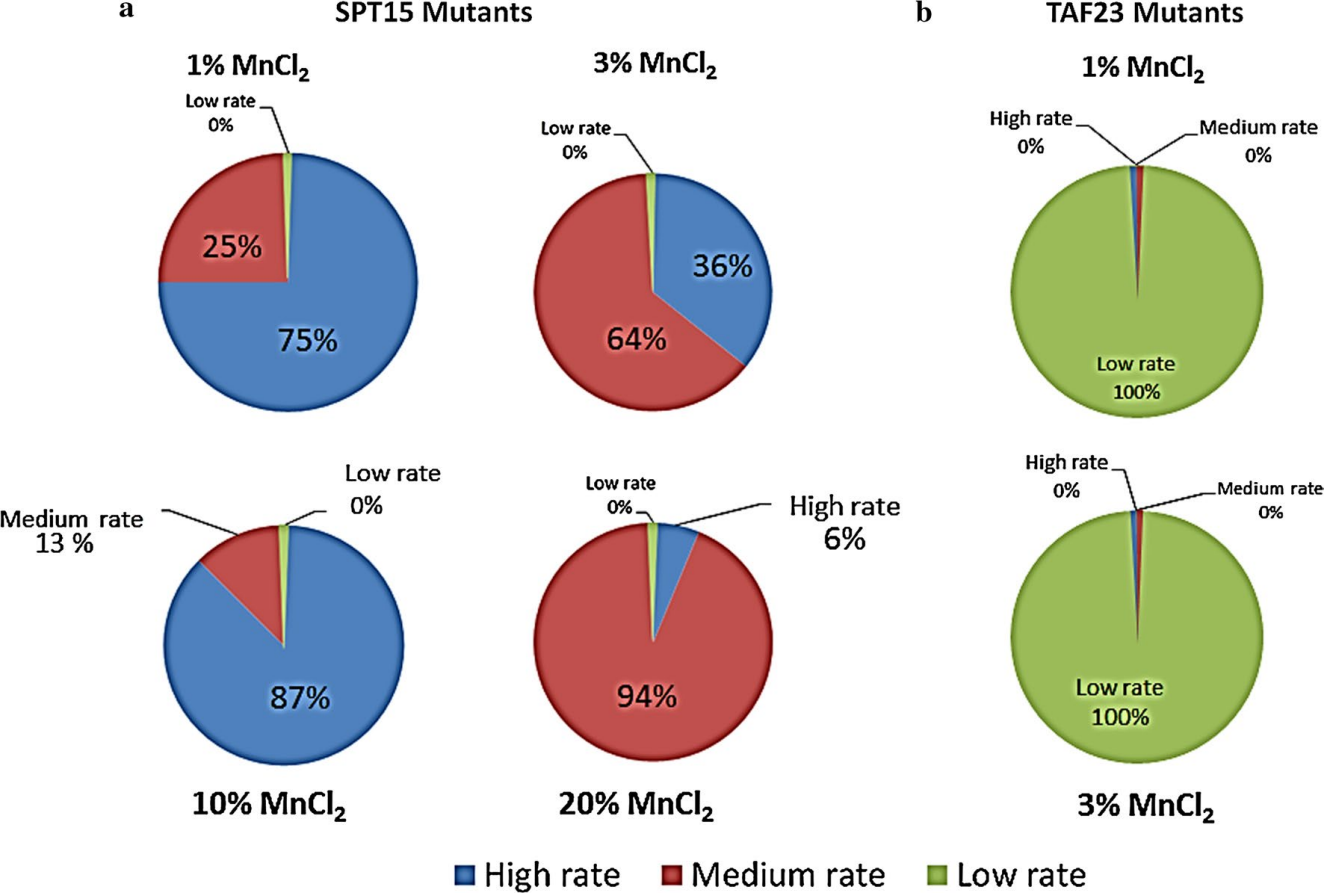
**a**, **b** Summary of the mutation rate for *SPT15* and *TAF23* mutant genes

The Ep-PCR was applied using different concentrations of MnCl_2_ (1, 3, 10 and 20%) for the alteration of each gene sequence, and 150 mutants were obtained. Based on the highest rate of success in the mutation, 50 mutants were chosen for *SPT15* and 6 mutants for *TAF23*. The chosen mutants were classified into three categories; (high, medium and low) based on the error rate (MER) according to the classification previously reported by Lanza and Alper (2011). Regarding the *SPT15* gene, 27 mutant strains were identified as medium MER and 23 mutant strains as high MER. In contrast, 6 mutant strains as low MER were detected with *TAF23* (Fig. 2a, b). All the strains have high similarity with the R-control strain which is at least >75%.

In details, by using 1% of MnCl_2_, 1 and 3 mutants with a medium and high MER, respectively were observed with *SPT15*; however 3 mutants with a low MER were observed with *TAF23*. Moreover, 3% of MnCl_2_ resulted in the construction of 5 and 9 mutants with a high and medium MER, respectively with *SPT15*. However 3 mutants with a low MER were constructed with *TAF23* using the same concentration. Furthermore, 14 mutant strains with a high MER and 2 mutant strains with a medium MER were formed by using 10% of MnCl_2_ with *SPT15*. Finally, 15 mutant strains with a medium MER and 1 mutant strain with a high MER were resulted by using 20% of MnCl_2_. No mutants were formed with *TAF23* by using both 10 and 20% of MnCl_2_.

### Construction of pYX212-SPT15-Mu and pYX212-TAF23-Mu plasmids

Both *SPT15*-*Mu* and *TAF23*-*Mu* genes were ligated individually with pYX212 using T4 DNA ligase to construct vector pYX212 with *SPT15* and *TAF23*, respectively. The resultants after ligation processes were named pYX212-SPT15-Mu and pYX212-TAF23-Mu, respectively. The gDNA *S. cerevisiae* R-control was used as a template for PCR reaction. The bands at 723 and 621 bp for *SPT15* and *TAF23* genes were identified, respectively after the plasmids were digested.

### Study of the ethanol tolerance of mutant strains

Five replicates of *TAF23* mutants were checked for the stability of G418 resistance gene, however they were not able to resist and to grow at the concentration 250 µg/mL of G418. In addition, 5 replicates of *SPT15* mutations by a total number of 250 were selected, and their stability tests were examined. These mutations were subjected to G418 at two different concentrations, 250 and 350 µg/mL, respectively (Walker et al. 2003), to screen the strains harboring stable resistance genes. Subsequently, the Normal PCR was conducted for these strains in order to amplify the successful resistance genes with concentrations of G418 to identify the extent of their activities and the stability for those mutations. Afterthought, we were able to choose the best 102 mutants with *SPT15* gene from the total selected 250 mutants. In addition, the total of 14, 24, 37 and 27 mutations were successfully chosen at 1, 3, 10 and 20% of MnCl_2_.

**Fig. 3:**
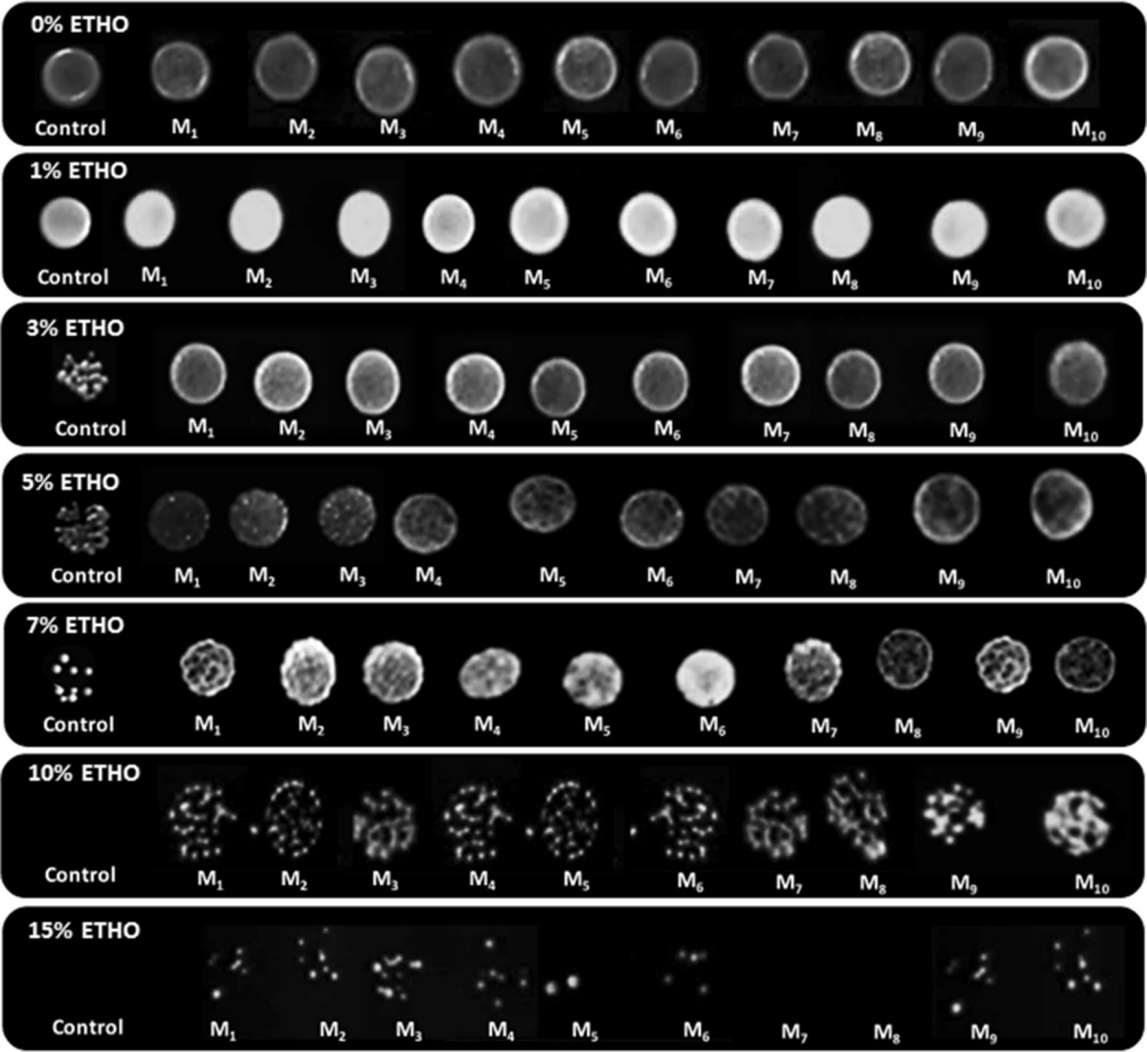
Susceptibility of ethanol tolerance of *S. cerevisiae* mutant strains with various levels of ethanol concentrations (0, 1, 3, 5, 7, 10 and 15%) on YPD media containing Kan. M1, M2, M3, M4, M5, M6, M7, M8, M9 and M10 refer to the numbering of mutations colonies; as follows: R-M20C1-P3, R-M16C4-P3, R-M4C1-P3, R-M12C4-P3, R-M18C4-P3, R-M5C1-P3, R-M3C49-P10, R-M18C5-P10, R-M17C4-P20 and R-M9C2-P20, respectively

The ethanol tolerance test was used to investigate 102 selected mutants. After 72 h of incubation at 30 °C, the colony formation was monitored and recorded. The transported colonies of the new mutants *SPT15* gene were compared to R-control strain that showed a slight improvement to form the colony on the media containing 1, 3, 5 and 7% (v/v) of ETOH. Conversely, the formation and composition of mutant colonies were weak in presence of 10% ETOH compared to the low concentrations. Meanwhile, the media containing 15% (v/v) of ETOH did not display any growth of colonies (Fig. 3).

### Effect of ethanol concentration on the growth rate of mutant strains

**Fig. 4:**
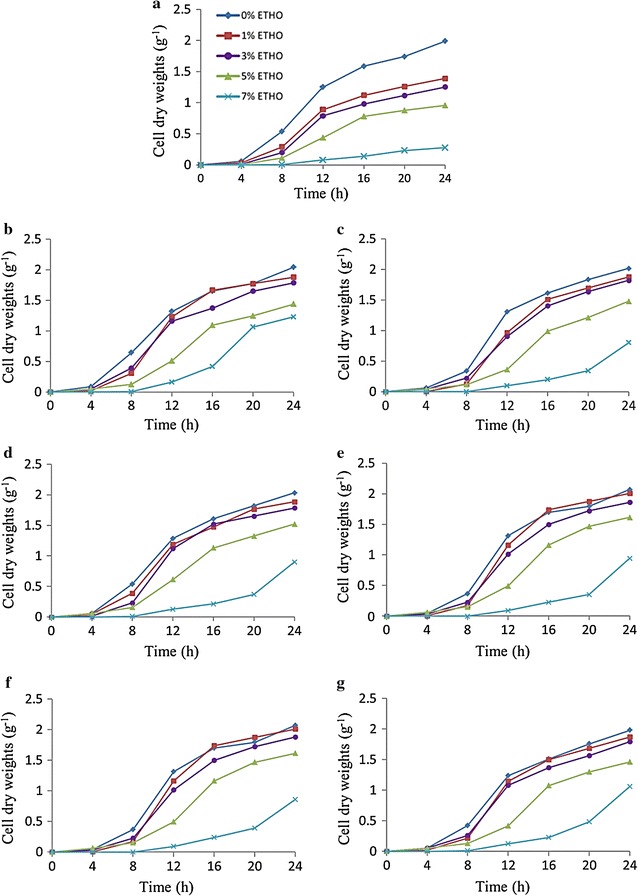
**a** Growth curve for the R-control strain and **b**–**g** growth curves for the best mutant strains (R-M20C1, R-M16C4, R-M4C1, R-M12C4, R-M18C4 and R-M5C1) obtained by the Ep-PCR reaction by using 3% MnCl_2_ concentration, respectively. These strains were inoculated into the YPDT medium at different levels of ethanol concentrations () 0% ETHO, () 1% ETHO, () 3% ETHO, () 5% ETHO and () 7% ETHO

**Fig. 5:**
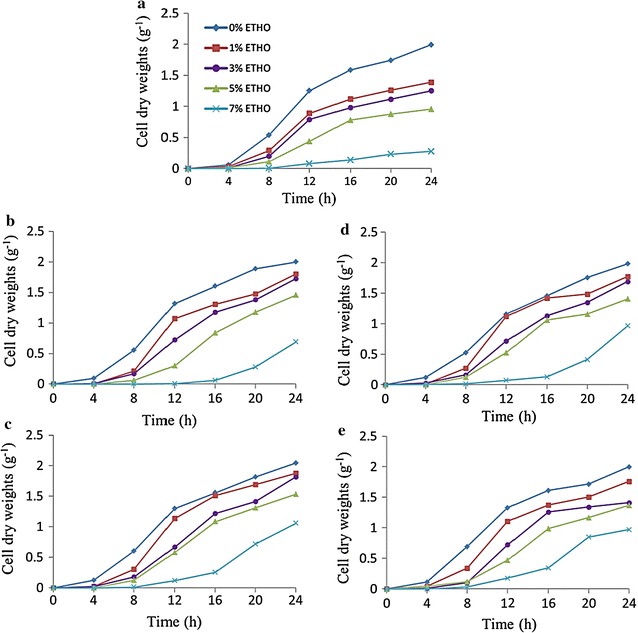
**a** Growth curve for the R-control strain and **b**–**e** growth curves for the best mutant strains (R-M3C49-P10, R-M18C5-P10, R-M17C4-P20 and R-M9C2-P20) obtained by the Ep-PCR reaction by using 10 and 20% MnCl_2_ concentrations, respectively. These strains were inoculated into the YPDT medium at different levels of ethanol concentrations () 0% ETHO, () 1% ETHO, () 3% ETHO, () 5% ETHO and () 7% ETHO

Results confirm of 94 transformants the previously mentioned stability tests, as the better growth rate for these mutants were observed when compared to the R-control strain (Figs. 4, 5). This result indicated that mutations contained *SPT15* genes possess the ability to grow coexisting with different concentrations of ethanol more than the R-control strain. Likewise, using a different concentration of MnCl_2_, the growth was better in the concentration of 3% followed by 10, 20 and 1%, respectively. It was shown that (R-M20C1-P3), (R-M16C4-P3), (R-M4C1-P3), (R-M12C4-P3), (R-M18C4-P3) and (R-M5C1-P3) recorded higher growth rates at 3% of MnCl_2_. As well, (R-M3C49-P10) and (R-M18C5-P10) were the highest growth mutants with MnCl_2_ 10%. Moreover, using 20% of MnCl_2_, (R-M17C4-P20) and (R-M9C2-P20) reported higher growth rates.

In details, mutants of R-M12C4-P3 and R-M18C4-P3 recorded a higher growth of values for DCW, which reached to 2.01 g/L (DCW) however the R-control recorded 1.39 g/L (DCW) at concentration of 1% ethanol. Moreover, the growth of R-M18C4-P3 mutant recorded the values for (DCW) 1.88 g/L at 3% ethanol; however 1.25 g/L was recorded for the R-control. On the other hand, the concentrations 5 and 7% of ethanol showed better growth rate for all mutants than R-control strain (Figs. 4, 5).

### Measurement of the ethanol and glucose yield

In order to estimate the efficiency of the different mutants for the production of ethanol, HPLC was used in this study. During the aerobic fermentation processes, the production of ethanol in the yeast expressing *SPT15* increased more than 60%, moreover the glucose decreased to 0. 19 g/L compared to the R-control strain. With the addition of 3% MnCl_2_, all the mutants showed higher ethanol production from 14.11 to 15.72 g/L compared to the R-control which produced 9.8 g/L, and the other used mutants with 1, 10 and 20% concentrations of MnCl_2_. The yield of the R-M20C1-P3 mutant strain reported the highest ethanol production 15.72 g/L with a reduction in glucose content 0.47 g/L. The production of ethanol was higher than R-control by using 1% of MnCl_2_, however decreased compared to the other mutants.

It is worth mentioning that the *SPT15* mutant library showed remarkable growth better than R-control strain in the presence of 1 and 3% ethanol with 20 g/L glucose. Consequently, the stress was increased to 7 and 10% ethanol in the subsequent serial sub-culturing. The reason for this phenomenon was speculated to be improved growth of all mutants due to their enhanced tolerance to ethanol stress, which was verified through higher mutants of cell density of R-M12C4-P3 and R-M18C4-P3 which recorded yield of 2.07 g/L (DCW) compare to R-control which was 1.99 g/L (DCW). This result was in accordance with previous results reported by Alper et al. (2006). Table 3 further summarizes results after anaerobic fermentation processes to make a comparison of parameters yields between the best mutant strains that obtained and R-control.

**Table 3:** Comparison of parameters yields of the best and highest mutant strains for ethanol production with R-control, during anaerobic fermentation in the presence of 20 g glucose/L

Strains^a^	Parameters					
	Final DCW (g/L)	Residual GLC (g/L)	Ethanol production (g/L)	Increase (%)	YEG ETHO g/GLC g	YEF ETHO g/CDW g
R-control	1.991	1.400	9.814	–	0.527	4.927
R-M20C1-P3	2.040	2.350	15.722	+60.3	0.891	7.706
R-M16C4-P3	2.014	1.900	15.471	+57.7	0.855	7.679
R-M4C1-P3	2.032	1.900	15.470	+57.7	0.855	7.616
R-M12C4-P3	2.071	1.750	15.322	+56.2	0.839	7.402
R-M18C4-P3	2.070	2.050	15.290	+55.9	0.852	7.387
R-M5C1-P3	1.981	1.700	15.211	+55.1	0.831	7.680
R-M3C49-P10	2.011	1.900	14.036	+43.1	0.786	7.029
R-M18C5-P10	2.040	2.150	14.008	+42.8	0.785	6.861
R-M17C4-P20	1.984	2.050	13.486	+37.5	0.756	6.811
R-M9C2-P20	2.012	2.200	13.464	+37.2	0.761	6.724

P3: 3% of MnCl_2_, P10: 10% of MnCl_2_ and P20: 20% of MnCl_2_; that were used in the Ep-PCR reaction

*CDW* dry cell weight, *GLC* glucose, *YEG* yield of ETHO g/glucose used g, *YEF* yield of ETHO g/final CDW g

^a^Indicates to the highest mutant strains for ethanol production

## Discussion

Up to the present time, the gTME technique has been used for the construction of new mutant; however in our study we processed a new yeast strain for a library of novel mutants by using *SPT15* gene. Moreover, the gTME technique has been modified by using MnCl_2_ instead of the most applied mixture Ep-PCR reaction MgCl_2_. By using different concentrations of MnCl_2_ resulted in increasing the error rate throughout DNA synthesis (Pavlov et al. 2004). This modification by using this condition led to an excess of changes from)Adenine to Guanine) and (Thymine to Cytosine), which had been a significant impact on the error rate (Sinha and Haimes 1981).The frequency of mutation in Ep-PCR could be changed by adjusting PCR conditions that provided accessibility in operation for evolution experiments (Wang et al. 2006). The haploid wild-type strains of *S. cerevisiae* (*MAT*-a) and (*MAT*-*α*) have been mated to produce a new diploid strain of *S. cerevisiae* R-control. In the gTME technique, the TBP plays an important role in encoding with *SPT15* and *TAF23* genes in *S. cerevisiae*. The theoretical studies have supposed that *TAF23* gene is critical to TBP interactions which might due to the presence of *TAF23* gene series of helices and linkers (Luhe et al. 2011; Alper et al. 2006; Mal et al. 2004; Gegonne et al. 2001). Consequently, results yielded 6 mutations with *TAF23*-*Mu* gene and 50 mutations with *SPT15*-*Mu* gene.

The G418 resistance could simplify the selection of stable transfected cell lines that reflected the characteristics of pYX212-SPT15-Mu and pYX212-TAF23-Mu at the expression of the stabilized genes which were not lost over time. The higher concentrations of G418 (350 µg/mL) enhanced the stability of mutant colonies at adverse pressure conditions that produced higher copy of mutations number. These results are similar to the finding reported by Wang et al. (2004).

The influences of ethanol on the growth of the *SPT15*-*Mu* and R-control strains have been investigated through the OD and DCW measurements. They were shown that the growth of all mutants increased gradually with all the concentration of ethanol compare to R-control. Furthermore, *SPT15* gene with the addition of thiamin 200 mg/L to the medium culture resulted in improving the alcoholic fermentation by enhancing the growth of yeast cells (Kotarska et al. 2006). In the same way, numerous proof-of-concept researchers have confirmed the increasing of cellular tolerances through increasing the production of ethanol and glucose (Liu et al. 2010; Tan et al. 2016). In order to *SPT15* as an ensemble, it can provide increased stronger resistance to ethanol and glucose. In addition, *SPT15* interacts with TBP to increase its efficient to the SAGA-dependent promoters of many genes (Bhaumik and Green 2002). In conclusion, the genetic results revealed that the gTME technique could be an effective approach for construction of novel mutants under various external stresses. The gTME technique to *S. cerevisiae* has been performed to adapt its attitude towards higher concentrations of ethanol. All the examined mutants showed much better tolerance toward ethanol stress as compared to the R-control. The mutants resulted by using 3% of MnCl_2_ in the process of Ep-PCR recorded the highest ethanol production. In a prospective study, we seek to establish a basis for future industrial applications, through integrating the *SPT15* mutant alleles of two new mutant strains into the chromosomes, to enhance ethanol tolerance and survive with high concentration of ethanol in the media.

## Abbreviations

gTME: global transcription machinery engineering
*E. coli*: *Escherichia coli*
*S. cerevisiae*: *Saccharomyces cerevisiae*
VHG: very high gravity
Amp: ampicillin
Kan: kanamycin
G418: glycosid-418 resistance gene
LB: luria–bertani medium
SOB: super optimal broth medium
YPD: bacto-peptone glucose broth medium
TBP: TATA-binding protein
gDNA: genomic DNA
OD: optical density
FDB: fast digest buffer
PCR: polymerase chain reaction
Ep-PCR: error prone polymerase chain reaction
*MAT*- α: *MAT*-alpha
HPLC: high performance liquid chromatography analysis
MCS: mutant colonies which were selected
AM: absent mutations
ETHO: ethanol
GLC: glucose
CDW: dry cell weight
YEG: yield of ethanol gram/glucose used gram
YEF: yield of ETHO gram/final CDW gram
ERM: error rate at mutants
